# Tuberculous meningitis in children is characterized by compartmentalized immune responses and neural excitotoxicity

**DOI:** 10.1038/s41467-019-11783-9

**Published:** 2019-08-21

**Authors:** Ursula K. Rohlwink, Anthony Figaji, Katalin A. Wilkinson, Stuart Horswell, Abdul K. Sesay, Armin Deffur, Nico Enslin, Regan Solomons, Ronald Van Toorn, Brian Eley, Michael Levin, Robert J. Wilkinson, Rachel P. J. Lai

**Affiliations:** 1 Neuroscience Institute, Division of Neurosurgery, University of Cape Town, Cape Town, South Africa; 2 Wellcome Centre for Infectious Diseases Research in Africa, Institute of Infectious Disease and Molecular Medicine, University of Cape Town, Cape Town, South Africa; 3 The Francis Crick Institute, London, NW1 1AT UK; 4 Department of Medicine, University of Cape Town, Cape Town, South Africa; 5 Department of Paediatrics and Child Health, Stellenbosch University, Stellenbosch, South Africa; 6 Paediatric Infectious Diseases Unit, Department of Paediatrics and Child Health, University of Cape Town, Cape Town, South Africa; 7 Department of Infectious Disease, Imperial College London, London, W2 1PG UK; 8 Present Address: Genomics Core, MRC Unit The Gambia at LSHTM, Serrekunda, The Gambia

**Keywords:** RNA sequencing, Tuberculosis, Paediatric research

## Abstract

Tuberculous meningitis (TBM) is the most severe form of TB with high rates of mortality and morbidity. Here we conduct RNA-sequencing on whole blood as well as on ventricular and lumbar cerebrospinal fluid (CSF) of pediatric patients treated for TBM. Differential transcript expression of TBM cases are compared with healthy controls in whole blood and with non-TB cerebral infection controls in CSF. Whole blood RNA-Seq analysis demonstrates a distinct immune response pattern in TBM, with significant increase in both canonical and non-canonical inflammasome activation and decrease in T-cell activation. In ventricular CSF, a significant enrichment associated with neuronal excitotoxicity and cerebral damage is detected in TBM. Finally, compartmental comparison in TBM indicates that the ventricular profile represents brain injury whereas the lumbar profile represents protein translation and cytokine signaling. Together, transcriptomic analysis shows that disease processes differ between the periphery and the central nervous system, and within brain compartments.

## Abstract (web summary)

Tuberculosis meningitis (TBM) is a severe form of TB with limited treatment options. Here, the authors perform RNA sequencing on whole blood and on ventricular and lumbar cerebrospinal fluid (CSF) samples from pediatric patients treated for TBM to characterize the immune response and tissue damage.

## Introduction

Tuberculosis remains a major contributor to the global disease burden^1^. Dissemination to the brain results in tuberculous meningitis (TBM), which is the most severe form of TB and leads to high rates of mortality and morbidity in both adults and children^2–4^. An important determinant of poor outcome is the host inflammatory response, which results in the formation of a thick exudate at the base of the brain. The exudate precipitates cerebral ischemia and infarction by causing vasculitis and occlusion of the major cerebral arteries and by disrupting the flow of cerebrospinal fluid (CSF) causing hydrocephalus and raised intracranial pressure. Understanding the cerebral inflammatory response, therefore, has been a focus of research into the pathophysiology and treatment of TBM.

Hitherto, studies investigating the immune response in TBM have focussed largely on immune mediators like cytokines and chemokines, but poor outcome inconsistently associates with the concentrations of these mediators^5–7^. While adjunctive therapeutic intervention with corticosteroids improves short-term mortality, it does not reduce morbidity and has no significant influence on immune mediator concentrations^4,8^. Furthermore, research on serial concentrations of biomarkers of brain injury and inflammation in pediatric TBM demonstrated that the inflammatory mediator concentrations decreased in all patients within 3–4 weeks after starting treatment. However, markers of brain tissue injury continued to increase in patients who died^9^. In combination, this evidence suggests that a further characterization of the host immune response and the consequent secondary brain injury mechanisms set in motion is critical, and could illuminate novel avenues for host-directed TBM treatment. In this study we investigate the transcriptome of children with TBM at the site of disease to elucidate key immune and injury-related pathways. Our previous research has demonstrated the significant differences in biomarker profiles between the periphery and site of disease, and between compartments within the central nervous system^9^. Therefore, we conduct transcriptomic analysis on blood, lumbar CSF, and ventricular CSF.

## Results

### Patient characteristics

The complete patient demographics and clinical characteristics are summarized in Table 1. Twenty TBM cases were enrolled; median age was 3 years (min–max: 0.3–12). Definite TBM was confirmed in 8 of the 18 patients (44%) who had CSF sent for TB culture or GeneXpert Mtb/Rif assay (Xpert, Cepheid). In two patients CSF was not sent for TB investigations, one of these patients was TB positive on tracheal aspirate. Pulmonary TB was reported in 40% (*n* = 8), and liver TB in 5% (*n* = 1) of TBM patients. No drug resistance was detected. All patients had associated hydrocephalus (100%) and features on contrasted brain CT in keeping with possible TBM: basal enhancement was presented in all patients, tuberculomas were identified on admission or follow-up scans in 30% (*n* = 6) of patients, and infarcts were present on 80% (*n* = 16). The 12-month mortality rate was 15% (*n* = 3). The three sample types were not available in all patients; whole blood RNA was available in 15 of the 20 TBM patients and ventricular and lumbar CSF RNA were available in 12 of the 20 TBM patients.

**Table 1:** Demographic and clinical characteristics

Characteristic	TBM cases (*n* = 20)	Non-TB infection controls (*n* = 7)	Healthy controls (*n* = 24)
Demographic characteristics			
Age (years)	3 (0.3–12)	1.1 (0.1–12.1)	5.1 (0·3–12.9)
Sex—male	10 (50)	6 (85.7)	19 (79.2)
CSF Investigations			
Ventricular CSF			
Glucose (mmol/l)	2.7 (1.5–5.4)	1.7 (0.2–1.9)	
Chloride (mmol/l)	114 (105–131)		
Protein (g/l)	0.8 (0.4–3)	2.3 (0.7–3.6)	
Lymphocytes (/cu mm)	37 (0–140)	54 (5–440)	
Polymorphonuclear cells (/cu mm)	4 (0–32)	18 (1–815)	
* Lumbar CSF*			
Glucose (mmol/l)	1.2 (<0.2–3)		
Chloride (mmol/l)	107 (99–121)		
Protein (g/l)	2.3 (0.9–36.7)		
Lymphocytes (/cu mm)	109 (15–1785)		
Polymorphonuclear cells (/cu mm)	8 (0–33)		
Admission characteristics			
MRC Staging admission			
1	2 (10)		
2	7 (35)		
3	11 (55)		
Admission GCS	9 (5–15)		
Seizures	13 (65)		
Focal neurology^a^	12 (60)		
Meningism	9 (45)		
HIV infection (*n* = 19)^b^	0 (0)		
Mortality	3 (15)		
CSF Investigations			
TB Culture, GeneXpert or AFB positive (*n* = 18)^c^	8 (44)		
Probable TBM^d^	12 (66)		

Values are reported as median (min–max range) or number (percentage), MRC Staging refers to British Medical Research Council TBM severity staging, CSF chloride was not measured as part of clinical routine for the non-TB infection controls, healthy controls did not have CSF sampling. Normal CSF values in pediatrics: glucose 2.3–3.9 mmol/l, chloride 120–130 mmol/l, protein 0.2–0.8 g/l, zero polymorphonuclear cells, <10 lymphocytes/cu mm

^a^Focal neurology included aphasia, absence of pupillary response, paresis, and cranial nerve palsies

^b^HIV testing was only conducted in 19 patients, the 20^th^ patient was not tested but the patient’s mother tested negative

^c^CSF was sent for diagnostic testing in 18 patients, one of the remaining 2 patients tested positive for TB on tracheal aspirate

^d^According to the consensus diagnostic criteria^29^

Twenty-seven healthy blood controls were enrolled, three were excluded due to a positive Quantiferon result (IGRA-positive). The remaining 24 patients had a median age of 5 years (min–max: 0.3–12.9); 79% were boys (*n* = 19). Reasons for elective surgery included intracranial pressure monitoring (17%, *n* = 4), dorsal rhizotomy (13%, *n* = 3), fatty filum terminale (29%, *n* = 7), cranioplasty (4%, *n* = 1), surgery for hydrocephalus (26%, *n* = 6), cranio-cervical junction surgery (4%, *n* = 1), and craniosynotosis (4%, *n* = 1). These patients did not have an evidence of CNS inflammation at the time of sample collection.

The seven other infection (OI) controls had a median age of 1.1 years (min–max: 0.1–12.1), 86% male (*n* = 6). Two patients had bacterial meningitis (*Streptococcus pneumonia* *n* = 1, no growth on culture *n* = 1). Four patients had bacterial meningitis secondary to shunt infections due to methicillin-resistant *Staphylococcus aureus* (*n* = 1), *Escherichia coli* (*n* = 1), *Staphylococcus epidermidis* (*n* = 1) and *Pseudomonas* (*n* = 1). One patient had neurocysticercosis.

Kolomogoro–Smirnov test showed no significance in age distribution. Healthy controls and OI controls were predominantly male, but it is unlikely that sex has a significant influence on the pathophysiology or clinical picture of the cases or controls.

### Whole blood transcriptome shows distinct immune responses

To investigate the transcriptional responses of pediatric TBM at a systemic level, whole blood from 39 patients (24 healthy controls and 15 TBM cases; Supplementary Data 1) was used for RNA sequencing. We excluded OI from final data analysis, as only four whole blood samples were available, rendering the group inadequately powered.

**Fig. 1:**
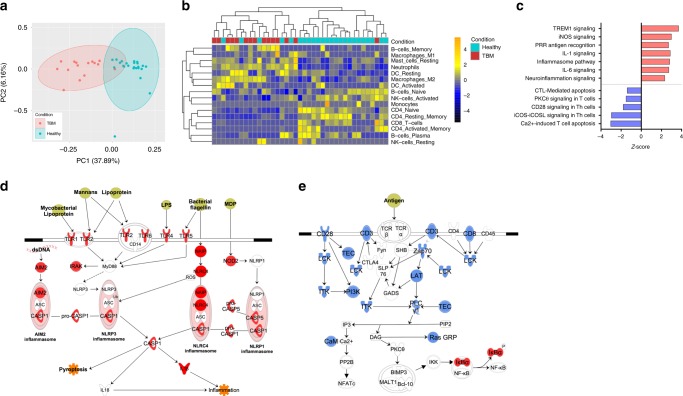
Whole blood transcriptome in pediatric TBM cases and IGRA-negative controls. **a** Principal component analysis on 2230 differentially expressed genes between TBM and healthy controls. **b** Deconvolution analysis revealed the in silico cellular composition of whole blood RNA. **c** Ingenuity canonical pathways overrepresented by differentially expressed genes are shown. The *z*-score indicates whether the pathway was upregulated (red) or downregulated (blue) in TBM. **d** Inflammasome signaling pathway is depicted. Molecules colored in red were significantly upregulated in TBM, compared to the controls. **e** T-cell activation pathway is shown. Molecules colored in blue were significantly downregulated in TBM, relative to the healthy controls

We identified 2230 genes that were differentially expressed between TBM cases and healthy controls using a stringent statistical filter (log2FC > 1 and padj < 0.05) (Supplementary Data 2). Principal component analysis on these differentially expressed genes showed distinct separation between the TBM cases and healthy controls (Fig. 1a). To identify cell components mediating the systemic transcriptional response, in silico deconvolution was performed using CIBERSORT with a validated leukocyte gene signature matrix^10^. Differential gene expression in TBM cases was driven predominantly by innate cell populations such as neutrophils, a known marker of pathogenesis in TBM, as well as macrophages, resting dendritic cells, and plasma B-cells (Fig. 1b). By contrast, gene expression in the healthy controls was predominantly associated with the CD4 (naïve, resting, and activated memory) and CD8 T-cells.

Ingenuity pathway analysis confirmed that the differentially expressed genes showed distinct immune responses in whole blood (Fig. 1c and Supplementary Data 3). When compared to the healthy controls, TBM cases showed significant upregulation in their innate immune responses. These include antigen recognition by pattern recognition receptors, inflammasome activation and IL-1 signaling, all of which are components of the proinflammatory inflammasome signaling pathways (Fig. 1d). The whole blood signature also reflects the disease site, as neuroinflammatory signaling was found to be upregulated in TBM. In line with the cell subset analysis, expression of genes over-representing T-cell activation and their signaling pathways was significantly downregulated in TBM (Fig. 1e).

We further performed sub-analyses to dissect the impact of several covariates and found that sex, TBM diagnosis (definite vs. probable) or TBM stage all had no influence in the whole blood transcriptomes. Age had a minor impact on the transcriptional responses in both TBM cases and healthy controls (0.47 and 0.16% of the transcriptome, respectively). The effect of age is also minute when considered as an interactive covariate in differential expression analysis comparing the two patient groups, as 2166/2230 genes remained significant and the functional pathways identified remained unchanged.

### Cerebral transcriptome reveals increased excitotoxicity

**Fig. 2:**
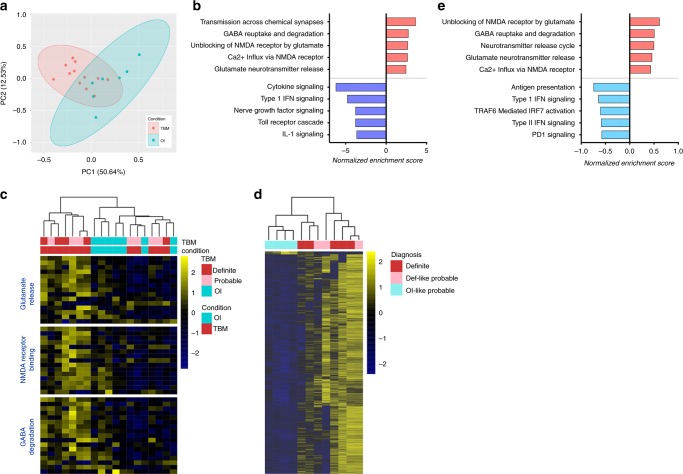
Transcriptome of ventricular CSF in pediatric TBM and other infection controls (OI). **a** Principal component analysis based on 312 differentially expressed genes between TBM and OI control is shown. **b** Reactome pathways enriched in TBM, relative to the controls, are listed. The normalized enrichment score indicates whether the pathway is positively enriched (red) or negatively enriched (blue) in TBM. **c** Heatmaps depicting the log2-transformed gene expression associated with glutamate release, NMDA receptor binding, or GABA degradation in the TBM group and the OI group. Each row represents one gene. **d** Heatmaps depicting the log2-transformed gene expression associated with glutamate release, NMDA receptor binding, or GABA degradation in relation to TBM diagnosis. **e** Reactome pathways enriched in OI-like probable TBM cases (blue), relative to definite or definite-like probable TBM cases (red) are listed

To better understand the cellular response in the brain, we profiled the global transcriptomes of ventricular CSF by sequencing. This was compared to the transcriptome of the non-TB OI controls. We did not directly compare the transcriptomic data from the CSF to those of the blood as the different library preparation used to accommodate for the low quantity (picogram range) of CSF RNA would compromise the compatibility of differential gene expression analysis. We analyzed ventricular CSF RNA-Seq data from 12 TBM cases (five definite and seven probable) and seven OI controls (Supplementary Data 4) and identified 312 genes that were differentially expressed (padj < 0.05) and which segregated the two groups by principal component analysis (Fig. 2a and Supplementary Data 5).

To understand the biological processes involved, we performed Gene Set Enrichment Analysis on the whole transcriptome pre-ranked on the Wald statistic. Immunological pathways such as those associated with cytokine signaling and toll-like receptor signaling were negatively enriched in TBM when compared with OI controls, with IL-1 and TNF being significantly downregulated (Fig. 2b and Supplementary Data 6). In addition, neutrophil-associated markers, such as S100A8, matrix metalloproteinases (MMP9/19/25) and tissue inhibitor of MMP (TIMP1) were all found to be significantly decreased in TBM. These inflammatory mediators have been found to be highly upregulated in blood or lumbar CSF of TBM^11–13^. In contrast, several pathways associated with glutamate neurotransmitter release, NMDA receptor binding and uptake were positively enriched in TBM (Fig. 2b), indicating that neuronal excitotoxicity was prominent in the ventricular CSF during TBM infection. Intriguingly, hierarchical clustering showed differential separation of patients with probable TBM (Fig. 2c). Post-hoc review found that the probable cases that cluster closely with definite TBM (definite-like) all had classical findings of TBM on brain imaging (e.g., precontrast basal hyperdensity, basal meningeal enhancement, typical pattern of tubercular infarcts, and/or tuberculomas). In contrast, three of the four probable cases that cluster with OI controls (OI-like) were found to have atypical imaging features (Supplementary Fig. 1).

To further explore the differential clustering among the seven probable TBM cases, subgroup analysis was performed to characterize gene expression between definite TBM (*n* = 5), definite-like probable TBM (*n* = 3), and OI-like probable TBM (*n* = 4). There was no transcriptional difference between definite TBM and definite-like probable cases, but significant transcriptional differences were consistently detected in 1815 genes (padj < 0.05), when comparing OI-like probables to definite TBM, as well as to definite-like probable cases (Fig. 2d and Supplementary Data 7). Gene Set Enrichment Analysis found that while definite and definite-like probable cases were enriched in pathways associated with glutamate neurotransmitter release, NMDA receptor binding, and GABA degradation, the OI-like probable cases were associated with immune response pathways such as antigen presentation, type I and type II interferon signaling and IRF7 activation (Fig. 2e and Supplementary Data 8).

Sub-analysis on the other covariates was also performed, but neither age, gender, mortality, nor stages of TBM had any significant impact on the CSF transcriptome. The results indicated that heterogeneity within all 12 TBM cases arose predominantly from those diagnosed with probable TBM, where there were two distinct subsets of patients.

### Distinct biological processes in lumbar and ventricular CSF

**Fig. 3:**
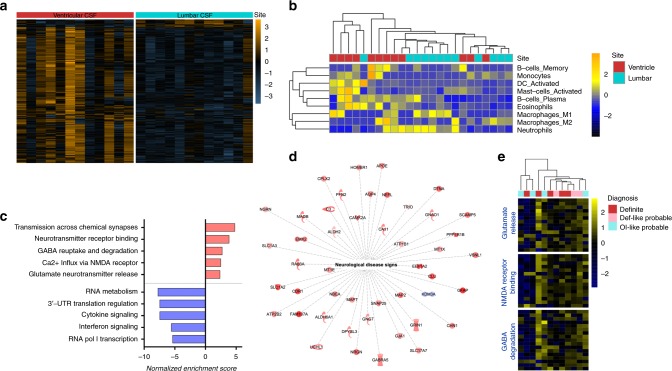
Transcriptome of ventricular CSF in comparison to lumbar CSF. **a** A heatmap depicting 389 differentially expressed genes between ventricular and lumbar CSF in pediatric TBM patients. **b** Deconvolution analysis revealed the in silico leukocyte composition of ventricular and lumbar CSF, respectively. **c** Reactome pathways enriched in ventricular CSF, relative to the lumbar are shown. The normalized enrichment score indicates whether the pathway is positively enriched (red) or negatively enriched (blue) in the ventricle of TBM patients. **d** Ingenuity disease network analysis clustered 46 of the differentially expressed genes into a network associated with neurological disease and injury. Molecules colored in red were significantly upregulated in ventricular CSF compared to lumbar CSF, while blue indicates significant downregulation. **e** A heatmap depicting the log2-transformed gene expression associated with glutamate release, NMDA receptor binding, or GABA degradation in relation to TBM diagnosis in lumbar CSF

We compared the transcriptomes between lumbar and ventricular CSF from our TBM cases (*n* = 12). Differential gene expression analysis identified 389 significantly different genes (Fig. 3a and Supplementary Data 9). In silico deconvolution on cellular composition with total transcriptomes revealed differences in leukocyte infiltration into the two different sites. Neutrophil transcripts were found to have predominantly higher presence in the lumbar CSF than in the ventricle (Fig. 3b). In contrast, other cellular subsets such as B-cells (memory and plasma), monocytes, and activated DCs, as well as nonneutrophil granulocytes were found in higher abundance in the ventricles.

Similar to ventricular CSF results above, Gene Set Enrichment Analysis revealed a strong positive enrichment in neuronal pathways, such as those associated with neurotransmitter release and neural tissue damage in ventricular CSF (Fig. 3c and Supplementary Data 9). In addition, increased transcription of genes associated with the potassium channel as well as nitric oxide stimulation were also identified (Supplementary Data 10). Disease network analysis on the 389 differentially expressed genes strongly indicated neurological disease and injury in the ventricle (*p* = 2.62 × 10^−13^, Fisher’s exact test), where 45/46 associated genes were upregulated (Fig. 3d). These 45 genes are mainly associated with glutamate excitotoxicity, a known mediator of many neurological diseases. In contrast, the lumbar CSF presented a substantially different transcriptomic landscape. Enhanced RNA transcription and protein translation, as well as cytokine signaling, were all overrepresented in lumbar CSF (Fig. 3c).

Given that probable TBM cases demonstrated two subgroups (definite-like and OI-like) in ventricular CSF, subgroup analysis was performed on the lumbar CSF transcriptomes. Surprisingly, no transcriptional difference was detected when comparing OI-like probable cases to definite TBM or to definite-like probable cases (Fig. 3e). Other co-variate analyses failed to find any significant impact of age, gender, stage of TBM or mortality on the transcriptome.

## Discussion

In this study we characterized the transcriptome of the whole blood, as well as two different sites of CSF (brain and spinal), to investigate disease processes occurring due to TBM. The main findings indicated a compartmentalized immune response in the peripheral blood and a gene signature overrepresented by neural injury in the CSF, particularly in the ventricular CSF. This is the first comprehensive analysis to date of both blood and CSF transcriptomes in TBM.

The pathogenesis of TBM is poorly understood due to the lack of an appropriate experimental model and the challenge of sampling the site of disease. Cohort studies in adults^14^ and in children^15^ have identified transcriptional signatures that could differentiate pulmonary TB from other infections, but whether these signatures can be applied for TBM remains to be investigated. A recent study used microarray to profile the whole blood transcriptomes of children with TBM at multiple time points and delineated reduced T-cell proliferation and immune responses over the course of disease progression^16^. While our study did not examine transcriptional changes over time, we identified a similar transcriptional reduction in peripheral T-cell responses when TBM cases were compared to IGRA-negative healthy controls. Intriguingly, a strongly upregulated innate immune response driven by inflammasome activation in whole blood was also identified. Our previous study on TBM-associated immune reconstitution inflammatory syndrome in adults also identified increased inflammasome signaling in the whole blood transcriptome and at protein level in the CSF^17^, further suggesting that inflammasome activation is a feature of TBM at the systemic level. In vitro and murine models of TB demonstrate that IFNγ and IFNγ-induced nitric oxide production are required to inhibit the NLRP3 inflammasome and the subsequent production of IL-1β to prevent neutrophil recruitment and tissue damage^18^. Thus, the lack of an effective T-cell response in pediatric TBM might be a contributing factor to the upregulated inflammasome activation.

Given the lack of access to brain tissues, we therefore utilized ventricular CSF as a proxy to study the cellular and immunological events in the brain. We have previously reported that ventricular CSF has significantly higher concentrations of cerebral injury markers relative to lumbar CSF^9^. This is in keeping with the cerebral site of production of these markers and the known rostro-caudal decrement in brain-derived proteins as CSF flows from the brain to the spinal canal^19,20^. Consistent with these data the transcriptomic signature in ventricular CSF revealed significant enrichment associated with neural excitotoxicity predominantly driven by glutamate release, NMDA receptor binding and uptake, features also associated with other forms of brain injury. Glutamate is a key excitatory neurotransmitter with important roles in learning and memory^21^. TBM-induced infarction and ischemia could potentially stimulate glutamate release^22^, leading to the excessive binding of glutamate to NMDA receptors on neurons. This precipitates an influx of Ca^2+^ and other positive ions into the neuron, resulting in the production of calpains, caspases, endonucleases, and phospholipases, and begins a cascade of injury mechanisms^23^. These include mitochondrial damage, breakdown of the cell membrane and cytoskeleton, production of nitric oxide and free radicals, acidosis, and DNA fragmentation, subsequently leading to apoptotic or necrotic cell death^24,25^. Our transcriptomic data also showed upregulation of genes associated with nitric oxide, cytochrome c, brain injury proteins like myelin basic protein, and proteins associated with neurodegenerative disease, including tau, amyloid-beta and apolipoprotein E. Excessive glutamate and neuro-excitotoxicity are thought to contribute to brain injury and cell death in both acute brain injury conditions, such as traumatic brain injury (TBI) and epilepsy, and chronic brain disorders such as Alzheimer’s and Huntington’s disease^26–28^. Excitotoxicity may increase the risk of clinical and subclinical seizures in TBM, and of cortical spreading depression, a secondary injury mechanism known to occur in stroke. These electrophysiological events increase metabolic demand on the already ischemic brain. Our data reflect the clinical picture of brain infarction in our TBM cases demonstrated on imaging, and begin to illuminate various brain injury mechanisms set up by the inflammatory response and the consequent ischemia.

Validation and targeted investigation of these excitotoxic mechanisms could identify novel therapeutic agents to ameliorate the ongoing brain injury. Recent data in mild TBI suggest chronic low-level neuroinflammatory processes may be initiated by minor insults like concussion and lead eventually to neurodegenerative disease if they occur repeatedly^24^. The considerable damage and inflammation precipitated by TBM may initiate similar long-term processes and poor outcomes. The differences between cases and controls indicate that it is unlikely that these findings simply reflect hydrocephalus or the method of ventricular puncture.

Intriguingly, on further examination of the excitotoxicity signature probable TBM cases differentiated into two subgroups: those with transcriptomic profiles more similar to the definite cases and those who were more closely related to OI controls. Posthoc analysis of their CT scans demonstrated differences between these two groups as well (Supplementary Fig. 1). Given that CSF culture positivity is low in most series, the diagnosis of TBM often relies on a suggestive history, clinical symptoms, CSF labs, and brain imaging findings. In the absence of a definitive “rule-out” test for TBM, it is not possible to determine whether probable cases are true TBM positive, or false positive. These data suggest that the transcriptional signature from ventricular CSF may be a possible way to identify different subgroups within the broad TBM diagnostic spectrum, thus avoiding unnecessary treatment and inappropriate inclusion in clinical trials. However, we caution that this hypothesis was generated from a small number of patients and posthoc analysis. Further prospective research would be needed to replicate and further explore this finding.

Although lumbar CSF is commonly used to study disease and treatment processes in TBM, our data on inflammatory and brain injury biomarkers in TBM demonstrate that ventricular CSF may be more representative of brain injury processes while lumbar CSF reflects inflammation. These compartmental differences are exemplified both at a clinical level in the chemistry data (Table 1) and also at transcriptional level. In silico deconvolution analysis in this study inferred a marked difference in cell composition between the lumbar and the ventricular space, where neutrophils were the dominant leukocytes in the lumbar CSF but showed low transcriptomic abundance in the ventricle. The predominance of monocytes in the ventricular CSF may reflect the local microglial response in the brain. Furthermore, the ventricular CSF neural injury signature was not present in the lumbar CSF, consistent with our biomarker work, which showed a greater concentration of cytokines and chemokines in the lumbar space and brain injury biomarkers in the ventricle^9^. Lumbar CSF did not show a transcriptional difference between definite and probable TBM cases, or within the probable group. This is likely because the transcriptional heterogeneity in probable TBM predominantly came from genes associated with excitotoxicity, which have low expression in lumbar CSF compared to ventricular CSF. Future larger studies with an increased sequencing read depth could improve the resolution of the transcriptional profiles in the lumbar CSF and may detect further heterogeneity within the TBM cases. These data highlight the importance of considering compartments in the central nervous system as distinct and evaluating ventricular CSF, when sampling is feasible, can offer novel insights into TBM pathogenesis.

We acknowledged that the small volume of CSF available in these young patients after clinical imperatives had been met limited the volume of CSF available for RNA extraction, especially since CSF cell counts are relatively low for RNA work. However, it is the first study to examine whole transcriptomics in the CSF of TBM patients, and offers insight into disease mechanisms to encourage further studies. The patients in this study all had hydrocephalus and the results in the absence of hydrocephalus may be dissimilar. However, it was by virtue of the need for CSF diversion that we were able to collect excess CSF for analysis, and the majority of pediatric TBM cases present with hydrocephalus. The non-TB infection control group presented with an acute rather than subacute inflammatory process (as exemplified in Fig. 2d), and shunt-related ventriculitis is not commonly associated with brain infarction. This may have contributed to the abundance of injury transcripts in the cases relative to controls; however, this was the most suitable diseased control group from whom we had access to sufficient CSF.

In conclusion, pediatric TBM appears to be characterized by an inflammasome-driven peripheral immune response, and brain injury appears to represent an important component of disease pathophysiology. There are important compartmental differences in TBM patients that are relevant to research studies. The disease pathways elucidated in this study offer valuable insight into potential targets for novel interventions that require further study.

## Methods

### Ethics

This study was approved by the scientific and human ethics review committees of the Faculty of Health Sciences of the University of Cape Town (HREC 200/2014). Parents/guardians provided informed consent for all study patients. Children older than 7 years provided assent as per South African national guidelines.

### Study cohorts

Patients treated for probable or definite TBM admitted to the Red Cross War Memorial Children’s Hospital, Cape Town, between July 2014 and February 2016 were recruited. TBM diagnosis was based on the consensus research definition, which combines clinical, radiological, and laboratory characteristics^29^. One patient was recruited from Tygerberg Hospital, Cape Town. All patients received standard antimycobacterial and adjunctive corticosteroid treatment^30^. Associated hydrocephalus was treated according to our institutional protocol^31^. Clinical data included admission signs and symptoms, demographics, death within 12 months, and radiological outcome, collected from admission and follow-up computed tomographic and/or magnetic resonance imaging brain scans. TBM disease severity was categorized using the refined British Medical Research Council criteria: Stage I: Glasgow Coma Score (GCS) 15 with no neurological deficit, Stage IIa: GCS 15 with neurological deficit/GCS13-14 with/without neurological deficit, Stage IIb: GCS 10–12 with/without neurological deficit, Stage III: GCS < 10 with/without neurological deficits^32,33^. Two control groups were enrolled; first, a group of patients undergoing elective neurosurgery for noninfectious conditions (healthy controls) from whom we collected blood; second, patients with meningitis or ventriculitis of non-TB origin (non-TB infection controls) from whom we collected blood and ventricular CSF. Since these patients required neurosurgical intervention with ventricular rather than lumbar puncture, lumbar CSF was not sampled. In those patients in whom lumbar punctures were performed, the small volumes of CSF obtained were prioritized for clinical imperatives.

### Sample collection

Blood samples from cases and controls were collected during clinically indicated venepuncture on admission or at surgery. Lumbar CSF was collected from cases during routine lumbar punctures for diagnostic or treatment purposes. Ventricular CSF was collected from cases and non-TB infection controls during clinically indicated neurosurgical interventions including insertion of an external ventricular drain or ventriculoperitoneal shunt. All samples were collected before or within 48 h of the first dose of anti-tuberculous treatment. Where possible samples across compartments were time-linked. Healthy controls also had blood collected for QuantiFERON TB Gold (QIAGEN) analysis to ascertain TB exposure.

### RNA extraction and sequencing library preparation

Whole blood (1–2.7 ml) was collected in PAXgene Blood RNA Tubes (QIAGEN/BD Biosciences), and total RNA was extracted using the PAXgene Blood RNA Kit (QIAGEN, Venlo, Netherlands). QuantiFERON testing was conducted using the QuantiFERON ELISA kit (QIAGEN). CSF was prioritised for clinical investigations and the remaining fluid was stored in RNA preservation buffer at −80 °C until RNA extraction was performed. RNA was extracted using the Direct-zol™ RNA MiniPrep kit (Zymo Research, Irvine, CA). The quantity and quality of the extracted RNA were measured by the Qubit fluorometer and the Caliper LabChip system, respectively. RNA libraries for whole blood RNA were constructed using the Ovation Human Blood RNA-Seq Library Systems (NuGEN Technologies, Red Wood City, CA) where ribosomal and globin RNA were removed according to the manufacturer’s protocol. RNA libraries for CSF RNA were constructed using the Ovation SoLo RNA-Seq system (NuGEN Technologies) due to the low quantity available. The final libraries were assessed using TapeStation 2200 System (Agilent, Santa Clara, CA). All libraries were sequenced on Illumina Hi-Seq 4000 instrument with paired-end 100 cycle reactions.

### RNA-Seq data analysis

The quality of the sequencing fastq files was analyzed using FastQC (v0.11.5) and poor quality samples were excluded from further differential gene expression analysis. Sequence reads were adapter- and quality-trimmed using Trimmomatic (v0.36) before aligning to the human genome (Ensembl GRCh38 build 88) using STAR aligner (v2.5.2a). Gene expression was quantified using RSEM (v.1.2.29), and differential gene expression analysis was performed using DESeq2 (v1.20.0) with default parameters for whole blood and with gene filtering (threshold ≥1) for CSF. DESeq2 uses a generalized linear model to estimate log2 fold change (log2FC) between comparison groups and the Benjamini-Hochberg false discovery rate was applied for multiple testing corrections, resulting in an adjusted *p*-value (padj) for each gene per comparison. Gene Set Enrichment Analysis on the whole transcriptome was performed by preranking all genes to the Wald statistic and referenced to the Reactome biological pathway database. Functional canonical pathways and disease networks associated with the differentially expressed genes were identified using Ingenuity IPA (QIAGEN).

### Role of the funding source

The funders of this study were not involved in the study design, data collection, data analyses, data interpretation, or report preparation. The corresponding author had full access to all the data in the study and had final responsibility for the decision to submit for publication.

### Reporting summary

Further information on research design is available in the Nature Research Reporting Summary linked to this article.

## Supplementary information

Supplementary Information

Reporting Summary

Description of additional supplementary files

Supplementary Data 1

Supplementary Data 2

Supplementary Data 3

Supplementary Data 4

Supplementary Data 5

Supplementary Data 6

Supplementary Data 7

Supplementary Data 8

Supplementary Data 9

Supplementary Data 10

## Data Availability

All RNA-Seq data has been deposited to Gene Expression Omnibus with accession number SE111459 for whole blood data and GSE122377 for CSF data.

## Code availability

All computer codes used in the study follow the vignette of each analytical package and are publically accessible. No customized code was used.
