# Supplementary material for: Tuberculous meningitis in children is characterized by compartmentalized immune responses and neural excitotoxicity
Source: Nat Commun. 2019 Aug 21;10:3767. doi: 10.1038/s41467-019-11783-9 (PMC6704154; doi:10.1038/s41467-019-11783-9)
Supplement: Supplementary file 1 — Supplementary Information [file 41467_2019_11783_MOESM1_ESM.pdf]

Supplementary Figure 1

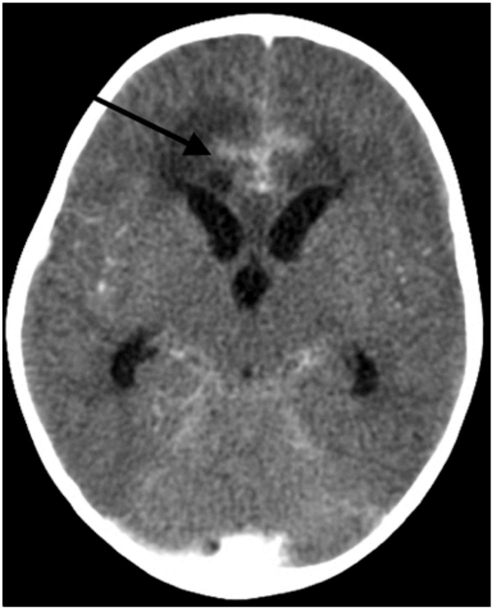

Contrasted Head CT scan showing hydrocephalas with enhancement in hte interhemispheric fissure and hydrodensities in the corpus callosum (arrowed). While compatible with the diagnosis of TBM in the presence of other criteria, these radiographic features are not considered classic.
