# Supplementary material for: Tuberculous meningitis in children is characterized by compartmentalized immune responses and neural excitotoxicity
Source: Nat Commun. 2019 Aug 21;10:3767. doi: 10.1038/s41467-019-11783-9 (PMC6704154; doi:10.1038/s41467-019-11783-9)
Supplement: Supplementary file 2 — Reporting Summary [file 41467_2019_11783_MOESM2_ESM.pdf]

nature research

Corresponding author(s): Rachel Pei Jen Lai

Last updated by author(s): Jun 11, 2019

Reporting Summary

Nature Research wishes to improve the reproducibility of the work that we publish. This form provides structure for consistency and transparency in reporting. For further information on Nature Research policies, see Authors & Referees and the Editorial Policy Checklist .

Statistics

For all statistical analyses, confirm that the following items are present in the figure legend, table legend, main text, or Methods section.

n/a	Confirmed	
☐	☒	The exact sample size (n) for each experimental group/condition, given as a discrete number and unit of measurement
☐	☒	A statement on whether measurements were taken from distinct samples or whether the same sample was measured repeatedly
☐	☒	The statistical test(s) used AND whether they are one- or two-sided<br>*Only common tests should be described solely by name; describe more complex techniques in the Methods section.*
☐	☒	A description of all covariates tested
☐	☒	A description of any assumptions or corrections, such as tests of normality and adjustment for multiple comparisons
☐	☒	A full description of the statistical parameters including central tendency (e.g. means) or other basic estimates (e.g. regression coefficient) AND variation (e.g. standard deviation) or associated estimates of uncertainty (e.g. confidence intervals)
☐	☒	For null hypothesis testing, the test statistic (e.g. F, t, r) with confidence intervals, effect sizes, degrees of freedom and P value noted<br>*Give P values as exact values whenever suitable.*
☒	☐	For Bayesian analysis, information on the choice of priors and Markov chain Monte Carlo settings
☐	☒	For hierarchical and complex designs, identification of the appropriate level for tests and full reporting of outcomes
☒	☐	Estimates of effect sizes (e.g. Cohen's d, Pearson's r), indicating how they were calculated

Our web collection on statistics for biologists contains articles on many of the points above.

Software and code

Policy information about availability of computer code

Data collection	RNA-Seq data were generated by Illumina Hi-Seq 4000 instrument using PE100 reaction
Data analysis	The quality of the sequencing fastq files was analysed using FastQC (v0.11.5) and poor quality samples were excluded from further differential gene expression analysis. Sequence reads were adapter- and quality- trimmed using Trimmomatic (v0.36) before aligning to the human genome (Ensembl GRCh38 build 88) using STAR aligner (v2.5.2a). Gene expression was quantified using RSEM (v.1.2.29) and differential gene expression analysis was performed using DESeq2 (v1.20.0) with default parameters for whole blood and with gene filtering (threshold above 1) for CSF.

For manuscripts utilizing custom algorithms or software that are central to the research but not yet described in published literature, software must be made available to editors/reviewers. We strongly encourage code deposition in a community repository (e.g. GitHub). See the Nature Research guidelines for submitting code & software for further information.

Data

Policy information about availability of data

All manuscripts must include a data availability statement. This statement should provide the following information, where applicable:

- Accession codes, unique identifiers, or web links for publicly available datasets
- A list of figures that have associated raw data
- A description of any restrictions on data availability

All RNA-Seq data has been deposited to Gene Expression Omnibus with accession number GSE111459 for whole blood data and GSE122377 for CSF data

Field-specific reporting

Please select the one below that is the best fit for your research. If you are not sure, read the appropriate sections before making your selection.

☒ Life sciences ☐ Behavioural & social sciences ☐ Ecological, evolutionary & environmental sciences

For a reference copy of the document with all sections, see nature.com/documents/nr-reporting-summary-flat.pdf

Life sciences study design

All studies must disclose on these points even when the disclosure is negative.

Sample size	Power was estimated using RnaSeqSampleSize package. The algorithm estimates power based on sample size, expected read counts and dispersion distribution. It controls for FDR associated with multiple testing. In our existing CSF RNA-seq data, we found a minimum of 250 DEG with >2 fold-change (assuming coverage of 10-12,000 genes with a minimum of 5 reads; FDR=0.05; dispersion effect=0.4) can consistently be detected when stratifying TBM patients.
Data exclusions	No exclusion criteria was applied in this study
Replication	Biological replicates are used in each patient arm for differential gene expression analysis
Randomization	No randomization was used - patient grouping is dependent on disease diagnosed
Blinding	Investigators are not blinded from the study

Reporting for specific materials, systems and methods

We require information from authors about some types of materials, experimental systems and methods used in many studies. Here, indicate whether each material, system or method listed is relevant to your study. If you are not sure if a list item applies to your research, read the appropriate section before selecting a response.

Materials & experimental systems

n/a	Involved in the study
☒	☐ Antibodies
☒	☐ Eukaryotic cell lines
☒	☐ Palaeontology
☒	☐ Animals and other organisms
☐	☒ Human research participants
☒	☐ Clinical data

Methods

n/a	Involved in the study
☒	☐ ChIP-seq
☒	☐ Flow cytometry
☒	☐ MRI-based neuroimaging

Human research participants

Policy information about studies involving human research participants

Population characteristics	Twenty TBM cases were enrolled; median age was 3 years (min-max: 0.3 - 12). Definite TBM was confirmed in 8 of the 18 patients (44%) who had CSF sent for TB culture or GeneXpert Mtb/Rif assay (Xpert, Cepheid). Twenty-seven healthy blood controls were enrolled, 3 were excluded due to a positive Quantiferon result (IGRA-positive). The remaining 24 patients had a median age of 5 years (min-max: 0.3 – 12.9); 79% were boys (n=19). The 7 other infection (OI) controls had a median age of 1.1 years (min-max: 0.1 – 12.1), 86% male (n=6). Two patients had bacterial meningitis (streptococcus pneumonia n=1, no growth on culture n=1). Four patients had bacterial meningitis secondary to shunt infections due to methicillin-resistant Staphylococcus aureus (n=1), Escherichia coli (n=1), Staphylococcus epidermidis (n=1) and Pseudomonas (n=1). One patient had neurocysticercosis.
Recruitment	Patients treated for probable or definite TBM admitted to the Red Cross War Memorial Children's Hospital, Cape Town, between July 2014 and February 2016 were recruited. TBM diagnosis was based on the consensus research definition, which combines clinical, radiological and laboratory characteristics.
Ethics oversight	This study was approved by the scientific and human ethics review committees of the Faculty of Health Sciences of the University of Cape Town (HREC 200/2014). Parents/guardians provided informed consent for all study patients. Children older than 7 years provided assent as per South African national guidelines.

Note that full information on the approval of the study protocol must also be provided in the manuscript.
