# Supplementary material for: Tuberculous meningitis in children is characterized by compartmentalized immune responses and neural excitotoxicity
Source: Nat Commun. 2019 Aug 21;10:3767. doi: 10.1038/s41467-019-11783-9 (PMC6704154; doi:10.1038/s41467-019-11783-9)
Supplement: Supplementary file 3 — Description of additional supplementary files [file 41467_2019_11783_MOESM3_ESM.pdf]

Description of Additional Supplementary Files

File Name: Supplementary Data 1
Description: Demographic and clinical characteristics of patients in whole blood transcriptome analysis

File Name: Supplementary Data 2
Description: List of 2230 differentially expressed genes between TBM cases and IGRA-negative healthy controls in whole blood

File Name: Supplementary Data 3
Description: Canonical pathways identified by Ingenuity Pathway Analysis in whole blood transcriptome of TBM

File Name: Supplementary Data 4
Description: Demographic and clinical characteristics of patients in ventricular CSF transcriptome analysis

File Name: Supplementary Data 5
Description: List of 312 differentially expressed genes between TBM cases and other infection (OI) controls in ventricular CSF

File Name: Supplementary Data 6
Description: Gene Set Enrichment Analysis (Reactome database) on ventricular CSF in TBM cases relative to other infection (OI) controls

File Name: Supplementary Data 7
Description: List of 1815 consistently differentially expressed genes between other infection (OI)-like probable TBM cases and definite TBM / definite-like probable TBM in ventricular CSF

File Name: Supplementary Data 8
Description: Gene Set Enrichment Analysis (Reactome database) on ventricular CSF in other infection (OI)-like probable TBM cases relative to definitely TBM / definite-like probable TBM

File Name: Supplementary Data 9
Description: List of 389 differentially expressed genes between ventricular CSF and lumbar CSF in TBM cases

File Name: Supplementary Data 10
Description: Gene Set Enrichment Analysis (Reactome database) on ventricular CSF relative to lumbar CSF in TBM cases
